# Non-pharmacological post-intensive care interventions to improve patient outcome following critical illness: a scoping review

**DOI:** 10.1186/s13054-025-05755-3

**Published:** 2025-11-28

**Authors:** Owen Gustafson, Rebecca Dalton, Neal Thurley, Natalie Pattison, Jonathan Bedford, Peter Watkinson, Sarah Vollam

**Affiliations:** 1 Critical Care Research Group, Nuffield Department of Clinical Neurosciences, University of Oxford, Oxford, UK; 2 Oxford Critical Care, Oxford University Hospitals NHS Foundation Trust, Oxford, UK; 3 Bodleian Healthcare Libraries, University of Oxford, Oxford, UK; 4 School of Health, Medicine and Life Sciences, University of Hertfordshire, Hatfield, UK; 5 East and North Herts NHS Trust, Stevenage, UK; 6 Imperial College London, London, UK; 7 NIHR Oxford Biomedical Research Centre, Oxford, UK; 8 Department of Critical Care, Milton Keynes University Hospital NHS Foundation Trust, Milton Keynes, UK

**Keywords:** Scoping review, Critical care, Recovery, Outreach, ICU follow-up

## Abstract

**Background:**

Patients discharged from intensive care units (ICU) commonly experience multiple problems in care during the acute hospital period. These can negatively impact their recovery and contribute to poor outcomes such as ICU readmission or in-hospital mortality. Many studies have aimed to address this through non-pharmacological interventions. However there has been no comprehensive synthesis of this literature. To improve care during the post-ICU in-hospital period, it is important to understand existing interventions and highlight priorities to guide future research. We therefore aimed to assess the extent of current literature relating to non-pharmacological interventions delivered to critical care survivors in hospital.

**Methods:**

We systematically searched five electronic databases (MEDLINE, EMBASE, CINAHL, AMED and CENTRAL) and grey literature to 4th February 2025. Search results were independently screened for eligibility by two reviewers at title, abstract and full text. Reports relating to non-pharmacological interventions delivered to adult patients in hospital following discharge from critical care were included. Study characteristics, intervention delivery and development, and outcomes were extracted using a formal data charting process.

**Results:**

Searches yielded 41,242 reports, from which 202 met the inclusion criteria. The most common interventions were critical care outreach/follow-up (CCOT/FU) (n = 93, 46%), physical rehabilitation (n = 77, 38.1%), and nutrition (n = 36, 17.8%). A small proportion of reports described randomised controlled trials (RCTs) (n = 31, 15.3%), of which few evaluated either CCOT/FU (n = 5, 16.1%) or multicomponent interventions (n = 6, 19.4%). Positive primary outcomes were reported by 51.6% (n = 16) of the RCTs (five of which were feasibility outcomes). Details of intervention implementation/training was included in only 17.6% (n = 18/102) of original studies. Interventions delivered by CCOT/FU included onward referrals, ordering investigations, family and patient support, and education provision.

**Conclusions:**

This review provides a comprehensive summary of the current knowledge of non-pharmacological interventions delivered following discharge from critical care, to improve outcomes. Although this is a large body of literature, it is limited by a lack of RCTs, and uncommon reporting of intervention development, implementation or training. Multicomponent interventions and CCOT/FU showed promising initial results but were infrequently evaluated in clinical trials, which should be considered for future investigation.

**Supplementary Information:**

The online version contains supplementary material available at 10.1186/s13054-025-05755-3.

## Background

Improvements in medical interventions, care delivery and technology have increased intensive care unit (ICU) survival rates significantly in recent years [1]. Despite this, every year one in ten patients discharged from ICUs in the United Kingdom (UK) either unexpectedly die in hospital or are readmitted to an ICU [2]. Problems in post-ICU care persist throughout the acute hospital period[2], contributing to these poor outcomes and leaving both patients and their families feeling vulnerable [3]. These outcomes occur despite strong evidence of avoidability [2, 4, 5] and presence of national guidance on providing in-hospital rehabilitation to maximise recovery since 2009 [6]. The recent UK National Confidential Enquiry into Patient Outcome and Death (NCEPOD) report has emphasised the need for improvement, highlighting significant shortfalls in rehabilitation provision in hospital following critical illness [7].

Many studies have associated factors at ICU discharge with poor patient outcome, including ongoing sepsis [2], tracheostomy presence [8] and high nursing workload [9]. These suggest that acute ward-based care following critical illness may not be adequate to manage ongoing organ dysfunction or high care needs. Other areas of poor care in-hospital delivery post ICU discharge have also previously been identified. These include the discharge process from an ICU [3], delivery of physical rehabilitation and nutrition [2, 5] and management of common medical problems [2]. However, the identification of these problems with in-hospital post-ICU care has not translated into interventions delivered in the acute hospital that meaningfully impact patient outcomes, despite critical care outreach (rapid response aimed at supporting deteriorating and at-risk patients in hospital) and in-hospital follow-up (supporting patients in the acute hospital ward following discharge from ICU) services being nationally recommended and commonly delivered [8, 10]. It is important to address these gaps in care urgently, as hospital discharge without a targeted in-hospital recovery strategy has profound adverse social and economic effects for patients and families [11, 12].

Multiple studies of non-pharmacological in-hospital interventions have attempted to address poor patient outcomes, however to date there has been no synthesis of this literature to help inform the development of future interventions. Therefore, this scoping review aims to assess the extent of the current literature relating to non-pharmacological interventions delivered during the acute hospital period to patients following discharge from critical care so that areas for future research to improve care can be identified. Additionally, within this body of literature, critical care outreach or in-hospital follow-up services (CCOT/FU) have commonly been referred to and evaluated as an intervention, although the term more accurately refers to a professional group delivering multiple varied interventions. For the purposes of this review, CCOT/FU will be referred to as an intervention to align with the literature, but the specific component interventions delivered by this group in each study will be extracted. Specifically, the review will: [1] report the extent of the literature evaluating non-pharmacological interventions aiming to improve physical and psychological outcomes delivered in hospital to patients following discharge from critical care, and [2] detail the interventions delivered by critical care outreach or follow-up services (CCOT/FU).

## Methods

### Protocol and registration

This scoping review was conducted in accordance with the JBI methodology for scoping reviews [13] and is reported in line with the Preferred Reporting Items for Systematic Reviews and Meta-Analyses extension for Scoping Reviews (PRISMA-ScR) guidelines [14]. The protocol was prospectively registered with the Open Science Framework on the 14th of November 2024 (10.17605/OSF.IO/2NPHT).

### Eligibility criteria

To be included in the review, papers needed to report or comment on non-pharmacological interventions delivered to adult patients in the acute ward setting which aimed to improve patient outcomes following discharge from critical care. To ensure the true extent of the literature was captured, published and unpublished studies of all methodologies were included if they were reported after 1999 and written in English. Studies including patients < 18 years or receiving specialist neurosurgical or cardiac critical care, or those evaluating a physical location (e.g. step-down unit) were excluded.

### Search strategy

We searched five databases for reports published between 1 st of January 2000 and 4th of February 2025. The databases searched were Cochrane Central Register of Controlled Trials (Cochrane Library, Wiley) [Issue 12 of 12, December 2024], AMED (OvidSP) [1985-February 2025], CINAHL (EBSCO) [1981-present], Embase (OvidSP)[1974-present], and Medline (OvidSP)[1946-present]. The WHO International Clinical Trials Registry Platform (https://trialsearch.who.int/), ProQuest Dissertations and Theses Global (https://about.proquest.com/en/products-services/pqdtglobal/) were also searched. The original search was run on Medline and EMBASE only in March 2024, with an updated search run on all five databases in May 2024 and then repeated in February 2025. The search strategies were developed by an experienced librarian (NT) and further refined through team discussion. The search comprised of title/abstract keywords and subject headings for post ICU hospitalised patients. This included prehospital discharge as well as ICU, intensive care or critical care in proximity to survivor, post, discharge or transition. No language limits were applied. For the second round of searching, publication years were limited to 1999 onwards. Reference lists were checked for those records that matched our inclusion criteria with forwards and backwards citation tracking also employed. The final search strategies are available in Additional File 1. Results were exported to Covidence (https://www.covidence.org/) where duplicates were removed both automatically and manually.

### Selection of sources of evidence

Two reviewers (OG and RD) independently screened the titles and abstracts for assessment against the inclusion criteria for the review. The full texts of potentially relevant sources were retrieved and independently assessed in detail against the inclusion criteria by the same two reviewers. Any disagreements that arose between the reviewers at each stage of the selection process were resolved initially through discussion, with a third reviewer (SV) available where consensus wasn’t achieved.

### Data charting and synthesis

The data extraction tool was developed by three reviewers (OG, SV and RD) and piloted independently by two reviewers (OG and RD) on three papers. The results were discussed in conjunction with a third reviewer (SV) and the tool was subsequently updated. Full details of the abstracted data are available in the Additional File 2. Descriptive statistics were used to tabulate and chart the collected data, and narrative synthesis used to analyse both the descriptions of the interventions undertaken in the studies and any training provided.

## Results

**Fig. 1:**
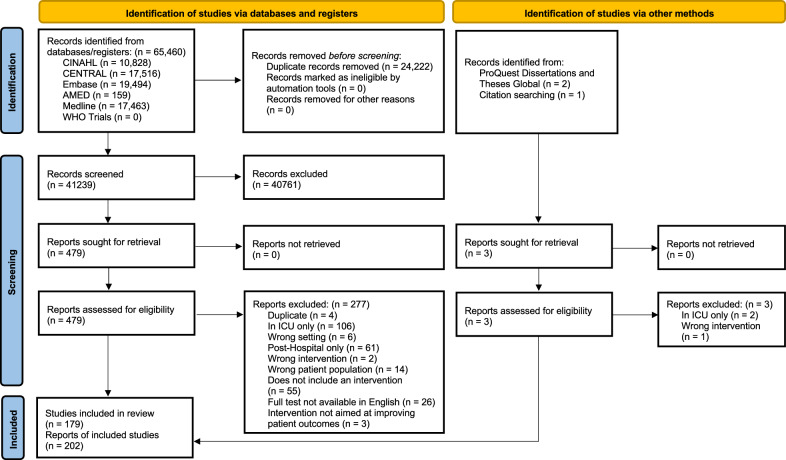
PRISMA flow diagram

Following the removal of duplicates, we identified 41,239 citations from searches of electronic databases and registers, and a further three citations from other methods (Fig. 1). We excluded 40,761 based on the title and abstract and a further 280 following a full text review. We included 202 reports of 179 individual studies in the review, the details of which are available in Additional File 1.

### Characteristics of sources of evidence

**Fig. 2:**
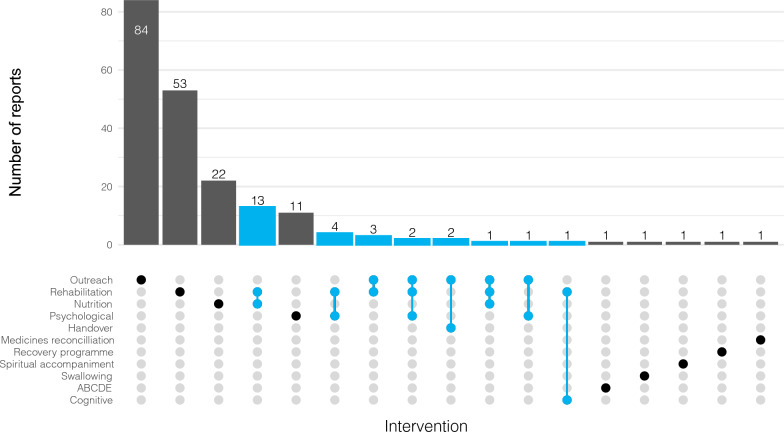
Interventions

Full details of the characteristics of the included reports are available in Additional File 2. Most reports originated from the UK (n = 42, 20.8%), USA (n = 34, 16.8%) and Australia (n = 33, 16.3%), with 45.5% (n = 92) published in the last five years. The primary interventions being investigated were CCOT/FU (n = 93, 46.0%), physical rehabilitation (n = 77, 38.1%) and nutrition (n = 36, 17.8%), with 13.4% (n = 27) investigating multicomponent interventions (Fig. 2). Reports were authored by a variety of professionals, with nurses (n = 77, 38.1%) and doctors (n = 68, 33.7%) most commonly first authors.

### Research methodologies

Table 1 outlines the types of reports included in our review. Most were of original research (n = 94, 46.5%) or literature reviews of varying methods (n = 52, 25.7%). Twelve study protocols (5.9%) and 15 (7.4%) study registrations were included in the review, of which three protocols and seven registrations relate to either ongoing or unpublished work. A small number of reports (n = 14, 6.9%) sought to qualitatively evaluate patient, family members and staff experiences and/or acceptability of the interventions being assessed [15–27], only one of which did not relate to CCOT/FU [27].

**Table 1:** Research methodologies of included reports

Type of report (n = 202)	N (%)
Original research	
Randomised controlled trial	31 (15.3)
Non-randomised experimental study	22 (10.9)
Case control	2 (1)
Case study	5 (2.5)
Cross sectional	3 (1.5)
Cohort	14 (6.9)
Qualitative	9 (4.5)
Mixed methods	5 (2.5)
Survey	2 (1)
Retrospective case record review	1 (0.5)
Literature reviews	
Systematic review	22 (10.9)
Scoping review	1 (0.5)
Integrative review	2 (1)
Narrative review	25 (12.4)
Protocols	
Study protocol	12 (5.9)
Study registration	15 (7.4)
Other	
Secondary analysis	1 (0.5)
Intervention development description	1 (0.5)
Service evaluation	8 (4)
Thesis	1 (0.5)
Conference abstract	8 (4)
Editorial/commentary	12 (5.9)

**Fig. 3:**
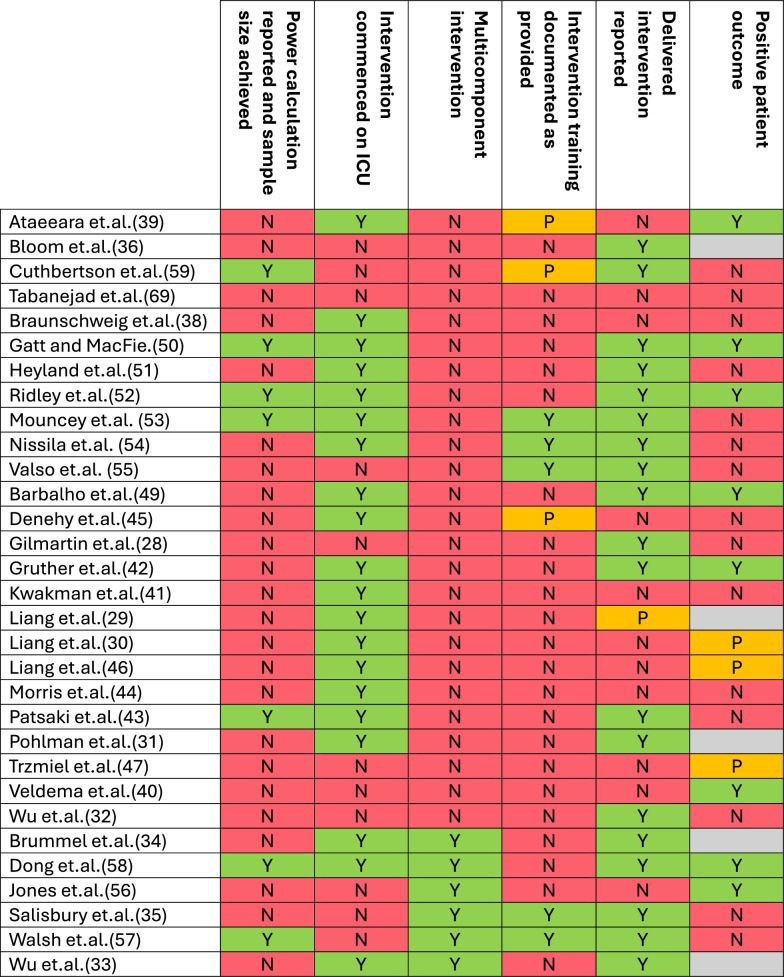
Randomised controlled trial outcome and characteristics Y = Yes, N = No, P = Partial, Blank = Feasibility outcome

Of the included experimental studies (n = 53, 26.2%), 58.5% (n = 31/53) were randomised controlled trials (RCTs) from 15 different countries (Table 2). Nine (29.0%) were pilot or feasibility trials [28–36] and 20 (64.5%) were single centre [28–30, 32–48]. Almost half of the included RCTs evaluated physical rehabilitation interventions alone (n = 14, 45.2%) [28–32, 40–47, 49], four (12.9%) exclusively evaluated nutrition interventions [38, 50–52] and three (9.7%) evaluated psychological interventions [53–55]. Only six studies (19.4%) included multicomponent interventions which consisted of physical rehabilitation with CCOT/FU [56], nutrition [33, 35, 57], psychological therapy [34], or cognitive rehabilitation [48] (Fig. 2). Of the studies which documented timing of instigation, eight began their interventions in ICU [30, 37, 39, 42, 46, 48, 49, 52], starting from 72 h following ICU admission [52] to 24 h prior to ICU discharge [58] (Fig. 3). There was wide variation in primary outcomes across the studies with several studies having multiple primary outcomes (Table 2). These could be categorised as physical function outcomes such as the Rivermead Mobility Index or Functional Independence Measure(n = 9) [28, 35, 40, 41, 45–47, 57, 59]; cognitive or psychological such as the Hospital Anxiety and Depression Scale(n = 6) [39, 53–56, 58]; intervention-specific outcomes such as handgrip dynamometry (n = 6) [30, 38, 43, 49, 50, 52]; and survival/length of stay outcomes such as post-ICU hospital length of stay (n = 5) [32, 42, 44, 51, 60]. A further five RCTs used primary outcomes related to intervention delivery or patient acceptability [29, 31, 33, 34, 36]. Statistically significant positive primary outcomes were reported by more than half of the RCTs (n = 16, 51.6%) [29–31, 33, 34, 36, 39, 40, 42, 46–50, 52, 56], however five (16.1%) of these reported on feasibility and deliverability outcomes [29, 31, 33, 34, 36] (Table 2 and Fig. 3). Of the 11 RCTs reporting a positive patient outcome two delivered a multicomponent intervention [48, 56], six delivered a physical rehabilitation intervention [30, 40, 42, 46, 47, 49], two delivered a nutrition intervention [37, 52], and one delivered a CCOT/FU intervention [39]. Of the seven studies which achieved sample sizes based on power calculations, three reported a positive outcome [37, 48, 52], one of which was multicentre [52]. These interventions included gut specific nutrients for critically ill surgical patients with inadequate gut function [37], a tailored nutrition programme delivered throughout hospitalisation [52], and a combination of physical and cognitive rehabilitation [48]. Full details of the methods employed in the included reports are available in Additional File 2. 

### Intervention development, training and documentation

Of the 102 papers reporting original research or service evaluations, 17 (16.7%) described the training provided to staff in order to deliver the intervention being evaluated [35, 39, 45, 53–55, 57, 59, 61–69]. The detail of intervention training described in the papers varied greatly from stating that all staff members involved attended a relevant course, to outlining the development of an education and training package including details on its delivery.

**Table 2:** Randomised controlled trials

Author, year, country	Ward-based intervention	Sample size, population, number of centres	Intervention training	Primary outcome, time	Primary results
Ataeeara et al., 2023, Iran[39]	CCOT/FU assessments and training for patients and families	200, Surgical ICU patients and their family, 1 centre	Two senior nurses received training on protocol implementation	Change in Spielberger state-anxiety questionnaire, 24 h after ICU discharge	The mean change of both patient (19.28 SD 11.22) and family (17.54 SD 10.52) anxiety state was significantly greater (p < 0.0001) than controls (0.54 SD 2.68 and—0.12 SD 3.35)
Bloom et.al., 2019, USA [36]	CCOT/FU inpatient visit	302, Medical ICU, 1 centre	Nil recorded	Number of components of the ICU recovery program intervention received, 30 days after hospital discharge	Patients in the intervention group received a median of two interventions compared with one intervention in the usual care group (p < 0.001)
Cuthbertson et.al, 2009, UK [59]	Manual based recovery programme	286, General ICU, 3 centres	Nursing staff followed a set format with standardised intervention and assessment requirements	SF-36, 12 months after randomisation	At 12 months, there was no evidence of a difference in the SF-36 physical component score (mean 42.0 (SD 10.6) v 40.8 (SD 11.9), effect size 1.1 (95% CI −1.9 to 4.2), p = 0.46) or the SF-36 mental component score (effect size 0.4 (−3.0 to 3.7), p = 0.83)
Tabanejad et.al., 2016, Iran [60]	ICU liaison nurse	85, General ICU, 2 centres	Nil recorded	Vital signs, satisfaction with care, post-ICU LOS, Hospital discharge	No significant differences between groups in vital signs, satisfaction in care, or post-ICU LOS
Braunschweig, et.al., 2015, USA [38]	Facilitated increased dietary intake	78, Acute lung injury patients from medical and surgical ICUs, 1 centre	Nil recorded	Rate of nosocomial infections, In hospital	No differences occurred in length of mechanical ventilation, hospital or ICU stay, or infections. The trial was stopped early because of significantly greater hospital mortality in the intervention group compared to control (40% vs 16%, p = 0.02)
Gatt and MacFie, 2010, UK [50]	Gut-specific nutrients	50, Surgical patients, 1 centre	Nil recorded	Time to return of normal gut function, calculated in hours to enteral tolerance of 80% of nutritional requirements for a continuous period of 48 h, In hospital	The intervention was associated with a quicker return of normal gut function (median 164 vs. 214 h; p = 0.016)
Heyland et.al, 2023, Canada [51]	High protein dose	1329, General ICU, 85 centres	Nil recorded	Time-to-discharge-alive from hospital, Hospital discharge	By 60 days after randomisation, the cumulative incidence of alive hospital discharge was 46.1% (95 CI 42.0%–50.1%) in the high-dose compared with 50.2% (46.0%–54.3%) in the usual dose protein group (hazard ratio 0.91, 95% CI 0.77–1.07; p = 0.27)
Ridley et.al., 2025, Australia [52]	Tailored nutrition	240, General ICU, 22 centres	Nil recorded	Energy delivered from nutrition therapy in kcal/day, Hospital discharge or day 28	Energy delivery was 1796 ± 31 kcal/ day (intervention) versus 1482 ± 32 kcal/day (control)—adjusted mean difference 271 kcal/day, 95% CI 189–354 kcal
Mouncey et.al., 2019, UK [53]	Stress support sessions, relaxation and recovery programme	787, General ICU, 24 centres	Comprehensive training programme including site visits, training courses, education, and skills development assessments	Symptom severity using the PTSD Symptom Scale – Self Report, quality-adjusted life-years and net monetary benefit, Six months	There was no difference in PTSD symptom severity between groups − 0.03, 95% CI − 2.58 to 2.52; p = 0.98. On average, the intervention decreased costs and slightly improved QALYs, leading to a positive incremental net benefit at 6 months (£835, 95% CI − £4322 to £5992), but with considerable statistical uncertainty surrounding these results
Nissila et.al., 2024, Finland [54]	Brief intervention for hazardous alcohol use	234, General ICU, 3 centres	Staff received training to deliver the brief interventions by an external trainer	Self-reported alcohol consumption converted to ethanol in grams, Six and 12 months after randomisation	There was no change in alcohol intake between intake in the intervention and control groups at six (p = 0.544) and 12 months (p = 0.157)
Valso et.al., 2020, Norway [55]	Nurse Led Follow-up consultations	523, Surgical and medical ICUs, 2 centres	Comprehensive training in crisis reactions and cognitive methods including case simulations and supervision from a psychiatrist and psychotruamatologist	Post-Traumatic Stress Scale 10 intensive part B score, Three, six and 12 months	No differences in post-traumatic stress symptoms or sense of coherence were found between intervention group versus control group
Barbalho et. al., 2019, Brazil [49]	Passive mobilization with blood flow restriction	20, General ICU, 2 centres	Nil recorded	Thigh muscle thickness and circumference, In hospital	The atrophy rate was lower in the blood flow restriction limb in relation to the control limb (2.1 vs 2.8 mm, respectively, in muscle thickness; p = 0.001)
Denehy et.al., 2013, Australia [45]	Cardiovascular, progressive resistance strength training and functional exercise	150, General ICU, 1 centre	All physical therapists working in the trial were required to have more than 2 years of clinical experience in the ICU. Written manuals of protocols and safety guidelines were supplied to all research personnel	6MWT, 12 months after ICU discharge	No significant differences were found for the primary outcome of 6MWT or any other outcomes at 12 months after ICU discharge
Gilmartin et.al., 2018, Ireland [28]	Rehabilitation after critical care assisted discharge pack	20, General ICU, 1 centre	Nil recorded	FIM, Spielberger state-anxiety questionnaire, Three weeks after ICU discharge	There was no significant difference in outcome between groups (p = 0.861)
Gruther et.al., 2017, Austria [42]	Early rehabilitation programme	53, Surgical and medical ICU, 1 centre	Nil recorded	Post-ICU LOS, Hospital discharge	Post-ICU LOS was 14 days (IQR, 12–20 days) in the early rehabilitation and 21 days (IQR, 13–34 days) in the standard-care group (p = 0.003)
Kwakman et.al., 2022, The Netherlands [41]	Bodyweight supported treadmill training	40, General ICU, 1 centre	Nil recorded	Days to achieve independent mobility defined by a Functional Ambulation Category of 3, In hospital	The median (IQR) time to independent ambulation was 6 (3 to 9) days in the intervention group (n = 19) compared to 11 (7 to 23) days in the usual care group (n = 21, p = 0.063)
Liang et.al, 2020, USA [29]	Exercise to rhythmic music	20, Medical, surgical, neurology and cardiovascular ICUs, 1 centre	Nil recorded	Bespoke intervention acceptability questionnaire, Five days after intervention or hospital discharge	Participants in the music group stated they found the exercise with rhythmic music intervention easier to understand (66.7% vs 33.3%), more motivating (55.6% vs 16.7%), more useful (66.7% vs 50%), and more enjoyable (77.8% vs 33.3%) than control participants
Liang et.al., 2022, USA [30]	Music-enhanced exercise intervention	26, Medical, surgical, neurology and cardiovascular ICUs, 1 centre	Nil recorded	Handgrip dynamometry, handheld dynamometry (elbow flexion, shoulder abduction, knee extension, plantar flexion), Hospital discharge	The intervention group had a statistically significant (p < 0.05) increase in bilateral knee extension and left plantar flexion strength compared to the control group
Liang et.al., 2023, USA [46]	Music-guided exercise intervention	26, Medical, surgical, neurology and cardiovascular ICUs, 1 centre	Nil recorded	PROMIS global health scale, in-hospital physical activity, intervention adherence, Hospital discharge	The intervention group showed significant improvement in perception of physical health compared to the active control group (6.5 ± 6.6 vs. − 1.62 ± 4.1, p = 0.017; Cohen’s d = 1.74). There was no difference in physical activity or intervention adherence
Morris et.al., 2016. USA [44]	Physical rehabilitation	300, Medical ICU, 1 centre	Nil recorded	Hospital LOS, Hospital discharge	The median hospital LOS was 10 days (IQR, 6 to 17) for the interventions group and 10 days (IQR, 7 to 16) for the usual care group (median difference, 0 [95% CI, − 1.5 to 3], p = 0.41)
Patsaki et.al., 2017, Greece [43]	Neuromuscular electrical stimulation	128, General ICU, 1 centre	Nil recorded	MRC SS and handgrip strength, Hospital discharge	MRCSS and handgrip strength did not differ at hospital discharge between groups (p > 0.05)
Pohlman et.al., 2010, USA [31]	Early physical and occupational therapy	49, Medical ICU, 2 centres	Nil recorded	Exercise intervention details, change in vital signs and ventilator asynchrony, Hospital discharge	Early physical and occupational therapy occurring a median of 1.5 days (range, 1.0–2.1 days) after intubation. Therapy was provided on 90% of medical ICU days during mechanical ventilation. At least one potential barrier to mobilization during mechanical ventilation was present in 89% of patient encounters
Trzmiel et.al., 2023, Poland [47]	Rehabilitation robot	86, COVID-19, 1 centre	Nil recorded	Tinetti scale, Berg scale, 6MWT, Muscle strength (elbow flexion/extension, handgrip), Barthel Index, FIM, Time not reported	The intervention group had a significantly greater improvement in FIM (p = 0.015) and mean elbow extensor strength (p = 0.023)
Veldema et.al., 2019, Germany [40]	Cycle ergometry and resistance exercises	39, General ICU, 1 centre	Nil recorded	Functional Ambulation Category, Four weeks after the intervention	A greater improvement in the Functional Ambulation Category was obtained by ergometer training, when compared to resistance training (p = 0.033)
Wu et.al., 2019, Australia [32]	In-reach mobile rehabilitation team	66, Surgical, medical, cardiac ICU, 1 centre	Nil recorded	Total hospital LOS, Hospital discharge	The total length-of-stay between the control and intervention groups (median 31 vs 41 days) was not statistically significant (p = 0.57)
Brummel et.al., 2014, USA [34]	Physical and cognitive therapy	87, General ICU, 1 centre	Nil recorded	Number of patients receiving cognitive therapy and details of the timing and content of the sessions, 12 months after hospital discharge	Early cognitive therapy was a delivered to 41/43 (95%) of cognitive plus physical therapy patients on 100% (92 −100%) of study days beginning 1.0 (1.0—1.0) day following enrolment. Physical therapy was received by 17/22 (77%) of usual care patients, by 21/22 (95%) of physical therapy only patients, and 42/43 (98%) of cognitive plus physical therapy patients on 17% (10—26%), 67% (46—87%), and 75% (59—88%) of study days, respectively
Dong et.al., 2023, China [58]	Cognitive and physical therapy	143, General ICU, 1 centre	Nil recorded	MoCA, Six months after enrolment	At six months the cognitive function score in the intervention group was significantly higher than that in the control group (26.69 ± 2.49 vs. 23.03 ± 3.79, p < 0.05)
Jones et.al., 2003, UK [56]	ICU Follow-up and rehabilitation manual	126, General ICU, 3 centres	Nil recorded	Spielberger state-anxiety questionnaire, HADS, IES, SF-36, Six months after ICU discharge	The intervention group demonstrated a greater improvement in SF-36 scores at 8 weeks and 6 months (p = 0.006) compared to the control group. There was no statistically significant difference in the other outcome measures
Salisbury et.al., 2010, UK [35]	Rehabilitation assistant delivered physical rehabilitation and nutrition	16, General ICU, 1 centre	Discipline-specific training was provided to ensure the generic rehabilitation assistant was competent to deliver the interventions	Rivermead mobility index, Timed up and go, 10 m walk test, incremental shuttle walk test, visual analogue scale, handgrip dynamometry, calorie and protein intake, Three months after ICU discharge	No statistically significant differences were found between groups for any of the outcome measures
Walsh et.al., 2015, UK [57]	Rehabilitation assistant delivered mobilisation, exercise, relevant dietetic therapy, Occupational Therapy, and Speech and Language Therapy	240, General ICU, 2 centres	The rehabilitation assistants received pretrial competency-based discipline specific training relevant to patients discharged from the ICU	Rivermead Mobility Index, Three months after randomisation	Median Rivermead Mobility Index at randomization was 3 (IQR, 1–6) and at 3 months was 13 (IQR, 10–14) for the intervention and usual care groups (mean difference, ‚−0.2 [95% CI, ‚−1.3 to 0.9; p =.71])
Wu et.al, 2024, China [33]	Nurse-driven Resistance Training with Hydroxymethylbutyrate	48, Medical, cardiac and respiratory ICU, 1 centre	Nil recorded	Recruitment rate, enrolment rate, retention rate, compliance rate, In hospital	All feasibility indicators met predetermined criteria. Forty-eight patients were randomly assigned across four arms, achieving a 96% enrolment rate. Most patients adhered to the intervention until discharge, resulting in a 97.9% retention rate. Compliance rates for both interventions approached or exceeded 85%

*CCOT/FU* = *critical care outreach or follow-up service, SD* = *standard deviation, SF-36* = *36-Item Short Form Survey, CI* = *confidence interval, LOS* = *length of stay, kcal* = *kilocalorie, PTSD* = *post-traumatic stress disorder, 6MWT* = *six-minute walk test, IQR* = *interquartile range, PROMIS* = *Patient-Reported Outcomes Measurement Information System, MRC SS* = *Medical Research Council Sum Score, FIM* = *Functional Independence Measure, MoCA* = *Montreal Cognitive Assessment, HADS* = *Hospital Anxiety and Depression Scale, IES* = *Impact of Events Scale*

Eight [35, 39, 45, 53–55, 57, 59] of the 31 RCTs included some description of intervention training that was provided as part of the study, however it is unclear if this had any influence on the outcomes of the study (Fig. 3). Additionally, twelve of the RCTs failed to clearly document the intervention that was delivered as part of the trial which makes it difficult to assess whether they delivered the planned intervention or not [29, 30, 38–41, 45–47, 56, 60]. The intervention development process was clearly documented in relation to a complex intervention framework by one trial and reported within several papers. This included a comprehensive description of the intervention development in relation to the Medical Research Council framework for developing and evaluating complex interventions [70], the study protocol [71], a feasibility study [35], a qualitative evaluation of the intervention [27], and the randomised controlled trial [57]. This programme of research evaluated generic rehabilitation assistants employed in the acute ward with the aim of improving patients’ mobility following critical illness. The final trial demonstrated no difference compared to control on mobility outcomes, however qualitative evaluation highlighted that patient perception of the intervention was positive.

###  Interventions delivered by critical care outreach or follow-up

A high number of papers included in the review reported on in-hospital CCOT/FU (n = 93), of which 49 were primary research. Three main titles were used to describe these services: ICU Liaison Nurse, Follow-Up Team, and Critical Care Outreach Team. Additionally two studies evaluated Rapid Response Teams [72, 73] and five evaluated specific tracheostomy services [74–78], all of which involved multidisciplinary post-ICU services and patients. Despite contributing a high proportion of the overall papers included in the review, interventions delivered by CCOT/FU were infrequently evaluated as part of an RCT (n = 5) [36, 39, 56, 59, 60]. Two RCTs reported a positive patient primary outcome [39, 56] and one reported a positive feasibility outcome [36]. Of these, only one RCT had a documented sample size informed by a power calculation which was met [59] and two studies described of any intervention training provided (although this was limited) [39, 59]. In terms of intervention timing and description, one of the trials with a positive outcome started their intervention in ICU, which consisted of a transition programme involving patient education, vital sign monitoring and introducing the patient to the ward staff [39]. However, there was no description of what parts of the intervention were successfully delivered in the trial. Two trials began their intervention on the acute ward and continued into the community [56, 59], both involving follow-up visits on the acute ward, provision of a recovery or rehabilitation manual, and subsequent outpatient clinic appointments. The studies had conflicting results, although neither described what was delivered to patients in the acute hospital. One study delivered their planned intervention exclusively in the acute ward, which involved a liaison nurse providing patient information, education, and support with self-care activities [60]. However, in common with most studies of CCOT/FU, there was no description of the intervention delivered. Only one reported trial provided details of the intervention, as feasibility of the intervention was the primary outcome [36]. This intervention included an ICU nurse visit on the acute ward where they provided patient education on recovery following critical illness and an education brochure. The patients also received a review by an ICU pharmacist who undertook medicines reconciliation. This was also the only trial to include a multidisciplinary service.

Of the 44 other primary research studies of in-hospital CCOT/FU, only 13 described the interventions they delivered on the acute ward [16, 19, 69, 73, 79–87], with two [16, 19] commenting only on the number of visits received by patients and not the type of interventions. The reported detail relating to the interventions and services delivered varied greatly among the remaining 11 studies. In these studies the teams predominantly consisted of critical care nurses with some services also including intensivists or physicians [73, 80, 81]. Where documented, patient eligibility criteria for the services were based on early warning scores, ward clinician judgement and referral, and/or patient ICU length of stay [81]. All services reported reviewing patients within 48 h of discharge from an ICU with patients receiving between one and three reviews on the acute ward [36, 69, 79, 80, 83, 85, 86]. Interventions reported as being delivered in these studies included: interprofessional referrals [69], ordering of investigations [69, 84], tracheostomy management [83, 84], medication administration [69, 83], family and patient support [83], ward staff education [79, 83], respiratory and cardiovascular interventions [79, 80, 83–85], mobilisation [80, 84, 85], and nutrition [85]. However, there were limited specific details of the interventions in relation to fidelity and dose achieved in the studies.

## Discussion

In this comprehensive scoping review, we have explored the extent to which non-pharmacological post-ICU interventions delivered in hospital have been reported and evaluated in order to characterise existing interventions and highlight priorities to guide future research. We identified 202 reports describing a variety of interventions including physical rehabilitation, nutrition, and outreach/follow-up. Our review illustrates the low number of experimental studies undertaken despite the high volume of published literature. Of the limited number of RCTs included in our review there was infrequent investigation of interventions delivered by CCOT/FU services or of multicomponent interventions. Details of the interventions being evaluated were limited, and very few demonstrated an impact on patient-centred outcomes.

Our results highlight potential areas of future investigation to improve care delivery to patients in hospital following discharge from an ICU. On the acute hospital ward following discharge from an ICU, numerous problems in care persist including challenges in rehabilitation and nutrition provision [2]. Given the increasing age and comorbidities of patients admitted to ICU [1], care provision on discharge from an ICU is likely to continue to be complex and demanding. To attempt to address these challenges, critical care outreach/in-hospital follow-up services have been in existence for many years and have been recommended in multiple clinical guidelines [10, 88]. This is emphasised by the large number of reports identified in our review (n = 93) that have evaluated these services. As critical care outreach/in-hospital follow-up services are frequently employed throughout the UK [8], they represent services that in-hospital post-ICU patients have in common and therefore could be the service which delivers future interventions to improve care delivery. However, the widespread adoption of critical care outreach/follow-up services has also made comprehensive investigation of this complex intervention challenging, as evidenced by our identification of only five [36, 39, 56, 59, 60] RCTs. In addition, studies commonly evaluated CCOT/FU as an intervention (with little description of what was delivered), rather than as a service which can deliver a variety of interventions. A future RCT of critical care outreach/in-hospital follow-up service delivered interventions will need to account for the wide variation in current clinical practice, service structure, workload, and wider roles within their organisation [23, 79, 87, 89]. We identified a variety of reports describing interventions focused on physical rehabilitation, psychological rehabilitation, and enhanced nutrition delivery which aim to address three of the major recovery challenges following discharge from an ICU. These problems can present in isolation; however, they frequently present in a variety of combinations as part of post-ICU syndrome, often persisting for years following an admission to ICU [11]. Specifically in the immediate period following discharge from an ICU, patients commonly experience a combination of these problems [90]. Despite this, most studies included in our review evaluated single interventions (Fig. 2). Several studies targeting specific aspects of recovery show some promise, demonstrating improvements in outcomes directly related to the intervention. Physical rehabilitation interventions have successfully shown impact on intervention specific outcomes such as thigh thickness [49], knee extension strength [30] and functional independence [40], while nutritional studies have significantly improved gut function [50] and energy delivered [52]. However, no RCTs to date have demonstrated significant improvement in broader patient outcomes such as hospital length of stay or survival. We found very few reports which evaluated multicomponent interventions, only six of which were RCTs [33–35, 48, 56, 57]. Given the heterogeneity of the ICU population and the multitude of problems encountered following discharge from ICUs, it is unlikely that interventions targeting a single aspect of care delivery is going to significantly alter patient outcome. Although the one included full RCT combining rehabilitation and nutrition interventions did not have a positive outcome, the success of some single-intervention trials does suggest a complex intervention targeting multiple areas of post-ICU rehabilitation could be beneficial to patient outcomes. Future studies evaluating care delivery in the acute hospital setting should focus on multicomponent interventions that can be adopted into the current available care pathways. When considering future studies, selection of appropriate outcome measures is crucial. Whilst trials assessing the impact on functional outcomes more commonly demonstrated a positive outcome, broader patient-level outcomes, such as hospital length of stay and survival, were much less commonly impacted. Future trials should carefully consider the primary outcome, ideally in collaboration with patients, to ensure the impact of interventions on patients-centred outcomes is captured. In particular, outcomes focused on mortality or hospital length of stay alone may not be relevant to patients, who put emphasis on the quality of their recovery [91]. Composite outcomes, such as hospital free days, are increasingly being used in trials, reflecting a focus on quality of recovery for patients [92]. One important observation from our review is that the interventions being reported can be considered complex and therefore should be developed using a complex interventions framework [93]. We found very few reports described the process of intervention development or even the intervention itself in any detail. Descriptions of the training delivered to staff for complex intervention delivery was also limited and often poorly described. For future interventions to be successfully adopted into clinical practice, they will need to be comprehensively developed and described, including staff training and intervention adherence [94].

### Strengths and limitations

Our review has several strengths. We developed the search strategy with an experienced medical librarian, searching multiple databases and strictly adhering to a prospectively registered protocol. We took a systematic and comprehensive approach, resulting in the identification of an extensive range of published and grey literature. Nevertheless, there are limitations to our review. Despite our comprehensive search, it did not include non-English language publications and may not have been exhaustive. However, it is unlikely that we missed any major interventions that are being employed and have been evaluated in the post-ICU in-hospital setting. Even though we deployed a data extraction tool that had several iterations and was piloted prior to instigation, it was difficult to extract accurate and complete data from all reports. There was great variation in the presentation and reporting of the methods and results of some included reports, therefore some data extraction was incomplete. We used the term CCOT/FU to encompass the wide variety of terminology and services employed to provide interventions to patients in an acute hospital following ICU discharge. However, it should be acknowledged that the primary role of some of these services (such as rapid response teams) is not focused on providing care to post-ICU patients. Finally, the scoping review methodology we employed is intended to provide a high-level mapping of the literature to inform research [95] and is not intended to comment on the quality of the included evidence [96].

### Recommendations for practice and future research

The recent publication of the NCEPOD report [7] on rehabilitation after critical illness highlights the urgent need to develop robust evidence-based interventions to support patients in the post-ICU in-hospital period, which is critical for recovery. Although we did not include any formal assessment of quality in this scoping review, recommendations for clinical practice from this review are restricted by the methodological and reporting limitations in most included studies. Most included RCTs were single site (limiting generalisability) and underpowered or not supported by a power calculation (limiting confidence in the result). In addition, broad and varied inclusion criteria were used making comparisons between studies and identification of the population who would most benefit from post-ICU interventions difficult to assess. However, multi-component studies and CCOT/FU interventions do show some potential benefit to patient outcomes. Future research should explore multi-component interventions that address multiple aspects of post-ICU recovery, as well as determining the optimal timing for these interventions, i.e. whether they should begin in the ICU, continue after hospital discharge, or both. Careful attention should be taken of adequate training to ensure the intervention is adhered to and delivered consistently, and to measuring this adherence and dose. Due to the nature of implementing a complex intervention into clinical practice, traditional trial designs will usually not allow for blinding of the intervention, and some cross-over into the control group is likely given the level of training required for successful implementation. Therefore, future studies should consider employing multi-site stepped wedge or cluster RCT designs to address this limitation. Additionally, future studies should make use of complex intervention development frameworks [93] to guide to successful trialling of interventions in such a complex area of care. Finally, it is essential that appropriate patient-centred outcomes which can feasibly be moderated by the intervention are selected.

## Conclusion

Our scoping review has synthesised a large body of literature evaluating non-pharmacological interventions delivered in-hospital following discharge from critical care, representing the best evidence to date. Despite the high number of included studies, we identified relatively few RCTs, and limited descriptions of intervention development, implementation, or training. The results of our review have identified areas with limited evaluation in clinical trials. These include both multicomponent interventions and interventions delivered by CCOT/FU, which should be considered for future investigation.

## Supplementary Information

Additional file 1.

Additional file 2.

## Data Availability

No datasets were generated or analysed during the current study.
