# Supplementary material for: Non-pharmacological post-intensive care interventions to improve patient outcome following critical illness: a scoping review
Source: Crit Care. 2025 Nov 28;30:1. doi: 10.1186/s13054-025-05755-3 (PMC12764170; doi:10.1186/s13054-025-05755-3)
Supplement: Supplementary file 1 — Additional file 1. [file 13054_2025_5755_MOESM1_ESM.docx]

Supplementary Material

Search strategy

*CINAHL*

| S1 | TI "pre hospital discharge" OR AB "pre hospital discharge" |
| --- | --- |
| S2 | TI "prehospital discharge" OR AB "prehospital discharge" |
| S3 | TI "critical illness" n3 surviv* OR AB "critical illness" n3 surviv* |
| S4 | TI discharg* n3 critical n3 survivor* OR AB discharg* n3 critical n3 survivor* |
| S5 | S1 OR S2 OR S3 OR S4 |
| S6 | TI ( ((icu or "intensive care" or "critical care") n3 (survivor* or post or discharg* or transfer* or transition*)) ) OR AB ( ((icu or "intensive care" or "critical care") n3 (survivor* or post or discharg* or transfer* or transition*)) ) |
| S7 | S5 OR S6 |
| S8 | (MH "Patient Discharge+") |
| S9 | (MH "Survivors+") |
| S10 | (MH "Transitional Care") |
| S11 | S8 OR S9 OR S10 |
| S12 | (MH "Intensive Care Units+") |
| S13 | (MH "Critical Care+") |
| S14 | (MH "Critical Illness") |
| S15 | S12 OR S13 OR S14 |
| S16 | S11 AND S15 |
| S17 | S7 OR S16 |
| S18 | S7 OR S16 |

*Cochrane Trials*

| #1 | pre hospital discharge:ti,ab,kw |
| --- | --- |
| #2 | prehospital discharge:ti,ab,kw |
| #3 | critical illness NEAR/3 surviv*:ti,ab,kw |
| #4 | discharg* NEAR/3 critical NEAR/3 survivor*:ti,ab,kw |
| #5 | #1 or #2 or #3 or #4 |
| #6 | ((icu or intensive care or critical care) NEAR/3 (survivor* or post or discharg* or transfer* or transition*)):ti,ab,kw |
| #7 | #5 or #6 |
| #8 | MeSH descriptor: [Patient Discharge] this term only |
| #9 | MeSH descriptor: [Survivors] this term only |
| #10 | MeSH descriptor: [Patient Discharge] this term only |
| #11 | MeSH descriptor: [Transitional Care] this term only |
| #12 | #8 or #9 or #10 or #11 |
| #13 | MeSH descriptor: [Intensive Care Units] explode all trees |
| #14 | MeSH descriptor: [Critical Care] this term only |
| #15 | MeSH descriptor: [Critical Illness] this term only |
| #16 | #13 or #14 or #15 |
| #17 | #12 or #16 |
| #18 | #12 or #16 with Publication Year from 1999 to 2024, in Trials |

*EMBASE*

| 1 | pre hospital discharge.ti,ab,kf. |
| --- | --- |
| 2 | prehospital discharge.ti,ab,kf. |
| 3 | (critical illness adj3 surviv*).ti,ab,kf. |
| 4 | (discharg* adj3 critical adj3 survivor*).ti,ab,kf. |
| 5 | 1 or 2 or 3 or 4 |
| 6 | ((icu or intensive care or critical care) adj3 (survivor* or post or discharg* or transfer* or transition*)).ti,ab,kf. |
| 7 | 5 or 6 |
| 8 | exp survivor/ |
| 9 | transitional care/ |
| \| 10 \| \| --- \| | 8 or 9 |
| 11 | exp intensive care/ |
| 12 | critical illness/ |
| 13 | 11 or 12 |
| 14 | 10 and 13 |
| 15 | 7 or 14 |
| 16 | conference*.pt. |
| 17 | 15 not 16 |
| 18 | limit 17 to yr="1999 -Current" |

*AMED*

| 1 | pre hospital discharge.ti,ab. |
| --- | --- |
| 2 | prehospital discharge.ti,ab. |
| 3 | (critical illness adj3 surviv*).ti,ab. |
| 4 | (discharg* adj3 critical adj3 survivor*).ti,ab. |
| 5 | ((icu or intensive care or critical care) adj3 (survivor* or post or discharg* or transfer* or transition*)).ti,ab. |
| 6 | 1 or 2 or 3 or 4 or 5 |
| 7 | patient transfer/ |
| 8 | survivors/ |
| 9 | patient discharge/ |
| 10 | 7 or 8 or 9 |
| 11 | exp critical care/ |
| 12 | critical illness/ |
| 13 | 11 or 12 |
| 14 | 10 and 13 |
| 15 | 6 or 14 |
| 16 | limit 15 to yr="1999 -Current" |

*Medline*

| 1 | pre hospital discharge.ti,ab,kf. |
| --- | --- |
| 2 | prehospital discharge.ti,ab,kf. |
| 3 | (critical illness adj3 surviv*).ti,ab,kf. |
| 4 | (discharg* adj3 critical adj3 survivor*).ti,ab,kf. |
| 5 | 1 or 2 or 3 or 4 |
| 6 | ((icu or intensive care or critical care) adj3 (survivor* or post or discharg* or transfer* or transition*)).ti,ab,kf. |
| 7 | 5 or 6 |
| 8 | Patient Transfer/ |
| 9 | Survivors/ |
| [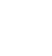](http://ezproxy-prd.bodleian.ox.ac.uk:2481/ovid-a/ovidweb.cgi?&S=GPKGFPOMHBEBKFBDIPAKNGEHIFAAAA00&R=3&Search+Annotations+Options=SA)   \| 10 \| \| --- \| | Patient Discharge/ |
| 11 | Transitional Care/ |
| 12 | 8 or 9 or 10 or 11 |
| 13 | exp Intensive Care Units/ |
| 14 | Critical Care/ |
| 15 | Critical Illness/ |
| 16 | **13 or 14 or 15** |
| 17 | 12 and 16 |
| 18 | 7 or 17 |
| 19 | limit 18 to yr="1999 -Current" |

Included references

1.Bloom S, Stollings J, Kirkpatrick O, Wang L, Byrne D, Sevin C, et al. 837: RANDOMIZED TRIAL OF AN INTENSIVE CARE UNIT RECOVERY PROGRAM FOR SURVIVORS OF CRITICAL ILLNESS. Critical care medicine. 2019;47:397–397.

2.Kheir F, Shawwa K, Nguyen D, Alraiyes AH, Simeone F, Nielsen ND. A 24-Hour Postintensive Care Unit Transition-of-Care Model Shortens Hospital Stay. Journal of intensive care medicine. 2016;31(9):597–602.

3.Hosey MM, Wegener ST, Hinkle C, Needham DM. A Cognitive Behavioral Therapy-Informed Self-Management Program for Acute Respiratory Failure Survivors: A Feasibility Study. Journal of clinical medicine [Internet]. 2021;10(4). Available from: <http://ovidsp.ovid.com/ovidweb.cgi?T=JS&PAGE=reference&D=pmnm6&NEWS=N&AN=33672672>

4.Brummel NE, Jackson JC, Girard TD, Pandharipande PP, Schiro E, Work B, et al. A combined early cognitive and physical rehabilitation program for people who are critically ill: the activity and cognitive therapy in the intensive care unit (ACT-ICU) trial. Physical therapy. 2012;92(12):1580–92.

5.Alberto L, Zotarez H, Canete AA, Niklas JEB, Enriquez JM, Geronimo MR, et al. A description of the ICU liaison nurse role in Argentina. Intensive & critical care nursing. 2014;30(1):31–7.

6.Ramsay P, Huby G, Rattray J, Salisbury LG, Walsh TS, Kean S. A longitudinal qualitative exploration of healthcare and informal support needs among survivors of critical illness: the RELINQUISH protocol. BMJ open [Internet]. 2012;2(4). Available from: <http://ovidsp.ovid.com/ovidweb.cgi?T=JS&PAGE=reference&D=pmnm2&NEWS=N&AN=22802422>

7.Eliott S, Chaboyer W, Ernest D, Doric A, Endacott R. A national survey of Australian Intensive Care Unit (ICU) Liaison Nurse (LN) services. Australian Critical Care. 2012;25(4):253–62.

8.Østergaard K. A New Model for Intensive Care Unit Follow-up: The ‘Attention-to’ List as an Alternative to the Checklist. CIN: Computers, Informatics, Nursing. 2023;41(4):195–204.

9.So HM, Yan WW, Chair SY. A nurse-led critical care outreach program to reduce readmission to the intensive care unit: A quasi-experimental study with a historical control group. Australian critical care : official journal of the Confederation of Australian Critical Care Nurses. 2019;32(6):494–501.

10.Samuelson KAM, Corrigan I. A nurse-led intensive care after-care programme - development, experiences and preliminary evaluation. Nursing in critical care. 2009;14(5):254–63.

11.Mouncey PR, Wade D, Richards-Belle A, Sadique Z, Wulff J, Grieve R, et al. A nurse-led, preventive, psychological intervention to reduce PTSD symptom severity in critically ill patients: the POPPI feasibility study and cluster RCT. 2019; Available from: <http://ovidsp.ovid.com/ovidweb.cgi?T=JS&PAGE=reference&D=medp&NEWS=N&AN=31465162>

12.Walsh TS, Salisbury LG, Boyd J, Ramsay P, Merriweather J, Huby G, et al. A randomised controlled trial evaluating a rehabilitation complex intervention for patients following intensive care discharge: the RECOVER study. BMJ open [Internet]. 2012;2(4). Available from: <http://ovidsp.ovid.com/ovidweb.cgi?T=JS&PAGE=reference&D=pmnm2&NEWS=N&AN=22761291>

13.Ramsay P, Salisbury LG, Merriweather JL, Huby G, Rattray JE, Hull AM, et al. A rehabilitation intervention to promote physical recovery following intensive care: a detailed description of construct development, rationale and content together with proposed taxonomy to capture processes in a randomised controlled trial. Trials. 2014;15:38.

14.Tabanejad Z, Pazokian M, Ebadi A. A Systematic Review of the Liaison Nurse Role on Patient’s Outcomes after Intensive Care Unit Discharge. International journal of community based nursing and midwifery. 2014;2(4):202–10.

15.Alberto L, Gillespie BM, Green A, Martinez MDC, Canete A, Zotarez H, et al. Activities undertaken by Intensive Care Unit Liaison Nurses in Argentina. Australian critical care : official journal of the Confederation of Australian Critical Care Nurses. 2017;30(2):74–8.

16.Barbalho M, Rocha AC, Seus TL, Raiol R, Del Vecchio FB, Coswig VS. Addition of blood flow restriction to passive mobilization reduces the rate of muscle wasting in elderly patients in the intensive care unit: a within-patient randomized trial. Clinical rehabilitation. 2019;33(2):233‐240.

17.Peters J, Aslakson R, Wilson M. Advanced Practice Registered Nurse--directed Care Coordination Interventions and Outreach After Critical Illness. Journal for Nurse Practitioners. 2018;14(6):e121–5.

18.Chillura A, Bramanti A, Tartamella F, Pisano MF, Clemente E, Lo Scrudato M, et al. Advances in the rehabilitation of intensive care unit acquired weakness: A case report on the promising use of robotics and virtual reality coupled to physiotherapy. Medicine. 2020;99(28):e20939.

19.Pattison NA, Dolan S, Townsend P, Townsend R. After critical care: a study to explore patients’ experiences of a follow-up service. Journal of clinical nursing. 2007;16(11):2122–31.

20.Merbitz NH, Westie K, Dammeyer JA, Butt L, Schneider J. After critical care: Challenges in the transition to inpatient rehabilitation. Rehabilitation psychology. 2016;61(2):186–200.

21.Endacott R, Eliott S, Chaboyer W. An integrative review and meta-synthesis of the scope and impact of intensive care liaison and outreach services. Journal of clinical nursing. 2009;18(23):3225–36.

22.Tobin AE, Santamaria JD. An intensivist-led tracheostomy review team is associated with shorter decannulation time and length of stay: a prospective cohort study. Critical care (London, England). 2008;12(2):R48.

23.Liu X, Long J, Chang Y, Gao H, Zhang X, Chen J, et al. Application of the whole-course care model (IWF/C Care) for postintensive care syndrome based on an early warning system in critically ill patients: a randomised controlled trial study protocol. BMJ open. 2023;13(7):e073035.

24.Wischmeyer PE. Are we creating survivors...or victims in critical care? Delivering targeted nutrition to improve outcomes. Current opinion in critical care. 2016;22(4):279–84.

25.Green A, Edmonds L. Bridging the gap between the intensive care unit and general wards-the ICU Liaison Nurse. Intensive & critical care nursing. 2004;20(3):133–43.

26.Odell M, Gerber K, Gager M. Call 4 Concern: patient and relative activated critical care outreach. British journal of nursing (Mark Allen Publishing). 2010;19(22):1390–5.

27.Gruther W, Pieber K, Steiner I, Hein C, Hiesmayr JM, Paternostro-Sluga T. Can Early Rehabilitation on the General Ward After an Intensive Care Unit Stay Reduce Hospital Length of Stay in Survivors of Critical Illness? A Randomized Controlled Trial. American journal of physical medicine & rehabilitation. 2017;36(5):607–15.

28.Wu J, Vratsistas-Curto A, Shiner CT, Faux SG, Harris I, Poulos CJ. Can in-reach multidisciplinary rehabilitation in the acute ward improve outcomes for critical care survivors? A pilot randomized controlled trial. Journal of rehabilitation medicine. 2019;51(8):598–606.

29.Chang AT, Boots RJ, Henderson R, Paratz JD, Hodges PW. Case report: inspiratory muscle training in chronic critically ill patients--a report of two cases. Physiotherapy research international : the journal for researchers and clinicians in physical therapy. 2005;10(4):222–6.

30.Carton E, Fitzgerald E, Elebert R, Malone C, O’Brien S, Dunne A, et al. Changes in Multidisciplinary Tracheostomy Team Practice Over Time. Irish medical journal. 2021;114(8):433.

31.Green A, Jones D, McIntyre T, Taylor C, Chaboyer W, Bailey M, et al. Characteristics and outcomes of patients reviewed by intensive care unit liaison nurses in Australia: a prospective multicentre study. Critical care and resuscitation : journal of the Australasian Academy of Critical Care Medicine. 2015;17(4):244–52.

32.Williams TA, Leslie G, Finn J, Brearley L, Asthifa M, Hay B, et al. Clinical effectiveness of a critical care nursing outreach service in facilitating discharge from the intensive care unit. American journal of critical care : an official publication, American Association of Critical-Care Nurses. 2010;19(5):e63-72.

33.Li Q., Yao L., Wang T., Liu Y. Construction and empirical of ICU patient follow-up model based on symptom management theory: a quasi-randomized controlled trial study protocol. medRxiv [Internet]. 2024;((Li, Yao, Wang, Wang) School of Nursing, Guizhou Medical University, Guizhou Province, China(Yao, Liu) School of Management, Guizhou University, Guizhou Province, Guiyang, China(Yao) Department of Respiratory and Critical Care Medicine, The Affiliated Hos). Available from: [https://www.medrxiv.org/https://ovidsp.ovid.com/ovidweb.cgi?T=JS&CSC=Y&NEWS=N&PAGE=fulltext&D=empp&DO=10.1101%2f2024.04.03.24305306https://oxford.primo.exlibrisgroup.com/openurl/44OXF_INST/44OXF_INST:SOLO?sid=OVID:embase&id=pmid:&id=doi:10.1101%2F2024.04.](https://www.medrxiv.org/https:/ovidsp.ovid.com/ovidweb.cgi?T=JS&CSC=Y&NEWS=N&PAGE=fulltext&D=empp&DO=10.1101%2f2024.04.03.24305306https://oxford.primo.exlibrisgroup.com/openurl/44OXF_INST/44OXF_INST:SOLO?sid=OVID:embase&id=pmid:&id=doi:10.1101%2F2024.04.)

34.Claffey A, Heslin C, Donnelly G, Charles R, Gorman S. Creating an integrated tracheostomy care pathway for patients in an Irish regional hospital setting - the feasibility and effectiveness of a dedicated tracheostomy team. International Journal of Integrated Care (IJIC). 2017;17:1–2.

35.Ball C. Critical care outreach services -- do they make a difference? Intensive & critical care nursing. 2002;18(5):257–60.

36.Niven DJ, Bastos JF, Stelfox HT. Critical care transition programs and the risk of readmission or death after discharge from an ICU: a systematic review and meta-analysis. Critical care medicine. 2014;42(1):179–87.

37.Stelfox HT, Bastos J, Niven DJ, Bagshaw SM, Turin TC, Gao S. Critical care transition programs and the risk of readmission or death after discharge from ICU. Intensive care medicine. 2016;42(3):401–10.

38.Osterlind J, Gerhardsson J, Myrberg T. Critical care transition programs on readmission or death: A systematic review and meta-analysis. Acta anaesthesiologica Scandinavica. 2020;64(7):870–83.

39.Veldema J, Bösl K, Kugler P, Ponfick M, Gdynia HJ, Nowak DA. Cycle ergometer training vs resistance training in ICU-acquired weakness. Acta neurologica Scandinavica. 2019;140(1):62‐71.

40.Rabiee A, Nikayin S, Hashem MD, Huang M, Dinglas VD, Bienvenu OJ, et al. Depressive Symptoms After Critical Illness: A Systematic Review and Meta-Analysis. Critical care medicine. 2016;44(9):1744–53.

41.Soo Hyun C, Jin Hee J, Sun Young W, Mi Sook O, Young Hee Y. Development of the transitional care program and its effect on patients discharged from the intensive care units: The World of Critical Care Nursing. 2015;9(4):145–145.

42.Taito S, Yamauchi K, Tsujimoto Y, Banno M, Tsujimoto H, Kataoka Y. Does enhanced physical rehabilitation following intensive care unit discharge improve outcomes in patients who received mechanical ventilation? A systematic review and meta-analysis. BMJ open. 2019;9(6):e026075.

43.Topaloglu M., Turan Z., Taskiran O.O. Early inpatient rehabilitation following intensive care unit discharge in acute respiratory distress syndrome due to Covid-19. Nobel Medicus. 2024;20(3):219EP – 223.

44.Vlake JH, van Bommel J, Wils EJ, Korevaar TI, Taccone F, Schut AF, et al. Effect of intensive care unit-specific virtual reality (ICU-VR) to improve psychological well-being in ICU survivors: study protocol for an international, multicentre, randomised controlled trial-the HORIZON-IC study. BMJ open. 2022;12(9):e061876.

45.Patsaki I, Gerovasili V, Sidiras G, Karatzanos E, Mitsiou G, Papadopoulos E, et al. Effect of neuromuscular stimulation and individualized rehabilitation on muscle strength in Intensive Care Unit survivors: A randomized trial. Journal of critical care. 2017;40:76–82.

46.Valso A, Rustoen T, Smastuen MC, Ekeberg O, Skogstad L, Schou-Bredal I, et al. Effect of Nurse-Led Consultations on Post-Traumatic Stress and Sense of Coherence in Discharged ICU Patients With Clinically Relevant Post-Traumatic Stress Symptoms-A Randomized Controlled Trial. Critical care medicine. 2020;48(12):e1218–25.

47.Nct. Effect of Protein, Mobility Therapy and Electric Stimulation on Recovery in Older ICU Survivors. https://clinicaltrials.gov/show/NCT05326633 [Internet]. 2022; Available from: <https://www.cochranelibrary.com/central/doi/10.1002/central/CN-02392495/full>

48.Ball C, Kirkby M, Williams S. Effect of the critical care outreach team on patient survival to discharge from hospital and readmission to critical care: non-randomised population based study. BMJ (Clinical research ed). 2003;327(7422):1014.

49.Haruna J, Unoki T, Nagano N, Kamishima S, Kuribara T. Effectiveness of Nurse-Led Interventions for the Prevention of Mental Health Issues in Patients Leaving Intensive Care: A Systematic Review. Healthcare (Basel, Switzerland) [Internet]. 2022;10(9). Available from: <http://ovidsp.ovid.com/ovidweb.cgi?T=JS&PAGE=reference&D=pmnm7&NEWS=N&AN=36141328>

50.Garcia-Perez-de-Sevilla G, Sanchez-Pinto Pinto B. Effectiveness of physical exercise and neuromuscular electrical stimulation interventions for preventing and treating intensive care unit-acquired weakness: A systematic review of randomized controlled trials. Intensive & critical care nursing. 2023;74:103333.

51.Al Chikhanie Y, Veale D, Schoeffler M, Pepin JL, Verges S, Herengt F. Effectiveness of pulmonary rehabilitation in COVID-19 respiratory failure patients post-ICU. Respiratory physiology & neurobiology. 2021;287:103639.

52.Choi S, Lee J, Shin Y, Lee J, Jung J, Han M, et al. Effects of a medical emergency team follow-up programme on patients discharged from the medical intensive care unit to the general ward: a single-centre experience. Journal of evaluation in clinical practice. 2016;22(3):356–62.

53.Wu CP, Xu YJ, Wang TG, Ku SC, Chan DC, Lee JJ, et al. Effects of a swallowing and oral care intervention for patients following endotracheal extubation: a pre- and post-intervention study. Critical care (London, England). 2019;23(1):350.

54.Dong Q, Yang Y, Tang Q, Yang M, Lan A, Xiao H, et al. Effects of early cognitive rehabilitation training on cognitive function and quality of life in critically ill patients with cognitive impairment: A randomised controlled trial. Australian Critical Care. 2023;36(5):708–15.

55.Chen Y, Wang R, Yu J, Zhu L, Lu Y, Deng X. Effects of MBSR therapy on negative emotions, fatigue, and sleep quality in ‘post-ICU patients’: A randomized controlled clinical trial protocol. Medicine. 2022;101(1):e28331.

56.Minetto MA, Fior SD, Busso C, Caironi P, Massazza G, Maffiuletti NA, et al. Effects of neuromuscular electrical stimulation therapy on physical function in patients with COVID-19 associated pneumonia: Study protocol of a randomized controlled trial. Contemporary clinical trials communications. 2021;21:100742.

57.Rosa RG, Ferreira GE, Viola TW, Robinson CC, Kochhann R, Berto PP, et al. Effects of post-ICU follow-up on subject outcomes: A systematic review and meta-analysis. Journal of critical care. 2019;52:115–25.

58.Rosseel Z., Cortoos P.-J., Leemans L., van Zanten A.R.H., Ligneel C., De Waele E. Energy and protein nutrition adequacy in general wards among intensive care unit survivors: A systematic review and meta-analysis. Journal of Parenteral and Enteral Nutrition. 2025;49(1):18EP – 32.

59.Marshall A.P., Ridley E.J., Chapple L.-A.S. Engaging family members in nutrition care during recovery from critical illness. Current Opinion in Clinical Nutrition and Metabolic Care. 2024;((Marshall) Intensive Care Unit, Gold Coast University Hospital, Southport, QLD, Australia(Marshall) School of Nursing and Midwifery, Griffith University, Southport, QLD, Australia(Ridley) Australian and New Zealand Intensive Care Research Centre, School o):10.1097/MCO.0000000000001100.

60.Ball C. Ensuring a successful discharge from intensive care. Intensive & critical care nursing. 2005;21(1):1–4.

61.Salt L. Evaluating critical care outreach and the early warning score tool – the ward nurse’s viewpoint. Kaitiaki Nursing Research. 2013;4(1):17–24.

62.Hang Mui S. Evaluation of the Effect of a Critical Care Follow-up Program on Patient Outcomes. Evaluation of the Effect of a Critical Care Follow-Up Program on Patient Outcomes. 2017;1–1.

63.Norwood MGA, Spiers P, Bailiss J, Sayers RD. Evaluation of the role of a specialist tracheostomy service. From critical care to outreach and beyond. Postgraduate medical journal. 2004;80(946):478–80.

64.Puthucheary ZA, Denehy L. Exercise Interventions in Critical Illness Survivors: Understanding Inclusion and Stratification Criteria. American journal of respiratory and critical care medicine. 2015;191(12):1464–7.

65.Connolly B, Salisbury L, O’Neill B, Geneen L, Douiri A, Grocott MPW, et al. Exercise rehabilitation following intensive care unit discharge for recovery from critical illness. The Cochrane database of systematic reviews. 2015;(6):CD008632.

66.Connolly B, Salisbury L, O’Neill B, Geneen L, Douiri A, Grocott MPW, et al. Exercise rehabilitation following intensive care unit discharge for recovery from critical illness: executive summary of a Cochrane Collaboration systematic review. Journal of cachexia, sarcopenia and muscle. 2016;7(5):520–6.

67.Denehy L, Skinner EH, Edbrooke L, Haines K, Warrillow S, Hawthorne G, et al. Exercise rehabilitation for patients with critical illness: a randomized controlled trial with 12 months of follow-up. Critical care (London, England). 2013;17(4):R156.

68.Nct. Exercise With Music for ICU Survivors. https://clinicaltrials.gov/show/NCT03885687 [Internet]. 2018; Available from: <https://www.cochranelibrary.com/central/doi/10.1002/central/CN-01919336/full>

69.Pattison N, Dolan S. Exploring patients’ experiences of a nurse-led follow-up service after critical care. Nursing times. 2009;105(19):16–9.

70.Siesage K, Joelsson-Alm E, Schandl A, Karlsson E. Extended physiotherapy after Intensive Care Unit (ICU) stay: A prospective pilot study with a before and after design. [Internet]. Philadelphia, Pennsylvania: Taylor & Francis Ltd; 2024 p. 1232–40. Available from: <https://go.openathens.net/redirector/nhs?url=https%3a%2f%2fsearch.ebscohost.com%2flogin.aspx%3fdirect%3dtrue%26AuthType%3dsso%26db%3dcin20%26AN%3d177396236%26site%3dehost-live%26profid%3Dehost>

71.Balas M, Buckingham R, Braley T, Saldi S, Vasilevskis EE. Extending the ABCDE bundle to the post-intensive care unit setting. Journal of gerontological nursing. 2013;39(8):39–51.

72.Liang Z, Munro CL, Ferreira TBD, Clochesy J, Yip H, Sena Moore K, et al. Feasibility and acceptability of a self-managed exercise to rhythmic music intervention for ICU survivors. Applied nursing research : ANR. 2020;54:151315.

73.Nct. Feasibility and Preliminary Effects of a Spiritual Care Strategy on Psychological Disorders in Critically Ill Patients. https://clinicaltrials.gov/ct2/show/NCT06048783 [Internet]. 2023; Available from: <https://www.cochranelibrary.com/central/doi/10.1002/central/CN-02599845/full>

74.Brummel NE, Girard TD, Ely EW, Pandharipande PP, Morandi A, Hughes CG, et al. Feasibility and safety of early combined cognitive and physical therapy for critically ill medical and surgical patients: the Activity and Cognitive Therapy in ICU (ACT-ICU) trial. Intensive care medicine. 2014;40(3):370‐379.

75.Pohlman MC, Schweickert WD, Pohlman AS, Nigos C, Pawlik AJ, Esbrook CL, et al. Feasibility of physical and occupational therapy beginning from initiation of mechanical ventilation. Critical care medicine. 2010;38(11):2089‐2094.

76.Kjer CKW, Estrup S, Poulsen LM, Mathiesen O. Follow-up after intensive care treatment: a questionnaire survey of intensive care aftercare in Denmark. Acta anaesthesiologica Scandinavica. 2017;61(8):925–34.

77.Defres S, Scott C, Park G. Follow-up ward rounds after intensive care--what do the patients and their visitors think? British journal of anaesthesia. 2005;95(6):837–8.

78.Liu K, Tronstad O, Flaws D, Churchill L, Jones AYM, Nakamura K, et al. From bedside to recovery: exercise therapy for prevention of post-intensive care syndrome. Journal of intensive care. 2024;12(1):11.

79.Carter CA. From ICU to outreach: A South African experience. Southern African Journal of Critical Care. 2008;24(2):50–5.

80.Strahan E, McCormick J, Uprichard E, Nixon S, Lavery G. Immediate follow-up after ICU discharge: establishment of a service and initial experiences. Nursing in critical care. 2003;8(2):49–55.

81.Garcea G, Thomasset S, McClelland L, Leslie A, Berry DP. Impact of a critical care outreach team on critical care readmissions and mortality. Acta anaesthesiologica Scandinavica. 2004;48(9):1096–100.

82.De Meester K, Das T, Hellemans K, Verbrugghe W, Jorens PG, Verpooten GA, et al. Impact of a standardized nurse observation protocol including MEWS after Intensive Care Unit discharge. Resuscitation. 2013;84(2):184–8.

83.Endacott R, Chaboyer W, Edington J, Thalib L. Impact of an ICU Liaison Nurse Service on major adverse events in patients recently discharged from ICU. Resuscitation. 2010;81(2):198–201.

84.Al-Qahtani S, Al-Dorzi HM, Tamim HM, Hussain S, Fong L, Taher S, et al. Impact of an intensivist-led multidisciplinary extended rapid response team on hospital-wide cardiopulmonary arrests and mortality. Critical care medicine. 2013;41(2):506–17.

85.Jensen JF, Thomsen T, Overgaard D, Bestle MH, Christensen D, Egerod I. Impact of follow-up consultations for ICU survivors on post-ICU syndrome: a systematic review and meta-analysis. Intensive care medicine. 2015;41(5):763–75.

86.Butcher BW, Vittinghoff E, Maselli J, Auerbach AD. Impact of proactive rounding by a rapid response team on patient outcomes at an academic medical center. Journal of hospital medicine. 2013;8(1):7–12.

87.van Mol M, Ista E, van Dijk M. Implementation and evaluation of a follow-up programme after intensive care treatment: A practice development project. Intensive & critical care nursing. 2018;49:6–13.

88.Villa M, Villa S, Vimercati S, Andreossi M, Mauri F, Ferlicca D, et al. Implementation of a Follow-Up Program for Intensive Care Unit Survivors. International journal of environmental research and public health [Internet]. 2021;18(19). Available from: <http://ovidsp.ovid.com/ovidweb.cgi?T=JS&PAGE=reference&D=med20&NEWS=N&AN=34639424>

89.Wibrandt I, Lippert A. Improving Patient Safety in Handover From Intensive Care Unit to General Ward: A Systematic Review. Journal of patient safety. 2020;16(3):199–210.

90.Hough CL. Improving physical function during and after critical care. Current opinion in critical care. 2013;19(5):488–95.

91.Nct. Improving Psychological Outcomes for Acute Respiratory Failure Survivors Using a Self-Management Intervention. https://clinicaltrials.gov/ct2/show/NCT06341972 [Internet]. 2024; Available from: <https://www.cochranelibrary.com/central/doi/10.1002/central/CN-02685217/full>

92.Cetto R, Arora A, Hettige R, Nel M, Benjamin L, Gomez CMH, et al. Improving tracheostomy care: a prospective study of the multidisciplinary approach. Clinical otolaryngology : official journal of ENT-UK ; official journal of Netherlands Society for Oto-Rhino-Laryngology & Cervico-Facial Surgery. 2011;36(5):482–8.

93.Walsh TS, Salisbury LG, Merriweather JL, Boyd JA, Griffith DM, Huby G, et al. Increased Hospital-Based Physical Rehabilitation and Information Provision After Intensive Care Unit Discharge: The RECOVER Randomized Clinical Trial. JAMA internal medicine. 2015;175(6):901–10.

94.Rodrigues M, Costa AJ, Santos R, Diogo P, Goncalves E, Barroso D, et al. Inpatient rehabilitation can improve functional outcomes of post-intensive care unit COVID-19 patients-a prospective study. Disability and rehabilitation. 2023;45(2):266–76.

95.Jonasdottir RJ, Klinke ME, Jonsdottir H. Integrative review of nurse-led follow-up after discharge from the ICU. Journal of clinical nursing. 2016;25(1–2):20–37.

96.Chaboyer W. Intensive care and beyond: improving the transitional experiences for critically ill patients and their families. Intensive & critical care nursing. 2006;22(4):187–93.

97.Gilmartin M, Moran F, Segurado R, O’Neill B. Intensive care discharge facilitation using the REhabilitation after Critical illness Assisted discharge Pack (RECAP) model: A pilot randomized controlled trial. Physiotherapy Practice & Research. 2018;39(1):63–74.

98.Barbetti J, Choate K. Intensive care liaison nurse service: implementation at a major metropolitan hospital. Australian Critical Care. 2003;16(2):46–52.

99.Burns C. Intensive care unit (ICU) outreach nurse: a literature review. Nursing Monograph. 2006;43–7.

100.Prevedello D, Fiore M, Creteur J, Preiser JC. Intensive care units follow-up: a scoping review protocol. BMJ open. 2020;10(11):e037725.

101.Braunschweig CA, Sheean PM, Peterson SJ, Gomez Perez S, Freels S, Lateef O, et al. Intensive nutrition in acute lung injury: a clinical trial (INTACT). JPEN Journal of parenteral and enteral nutrition. 2015;39(1):13‐20.

102.Nct. Intensive Nutrition in Critically Ill Adults. https://clinicaltrials.gov/show/NCT03292237 [Internet]. 2017; Available from: <https://www.cochranelibrary.com/central/doi/10.1002/central/CN-01564335/full>

103.Nct. INTENT-Muscle (A Sub-study of INTENT). https://clinicaltrials.gov/show/NCT04896515 [Internet]. 2021; Available from: <https://www.cochranelibrary.com/central/doi/10.1002/central/CN-02289569/full>

104.Calvo-Ayala E, Khan BA, Farber MO, Ely EW, Boustani MA. Interventions to improve the physical function of ICU survivors: a systematic review. Chest. 2013;144(5):1469–80.

105.Vegh LA, Blunt AM, Wishart LR, Gane EM, Paratz JD. Managing deteriorating patients with a physiotherapy critical care outreach service: A mixed-methods study. Australian critical care : official journal of the Confederation of Australian Critical Care Nurses. 2023;36(2):223–31.

106.Nct. Move to Music Video Intervention for ICU Survivors. https://clinicaltrials.gov/show/NCT04977297 [Internet]. 2021; Available from: <https://www.cochranelibrary.com/central/doi/10.1002/central/CN-02290983/full>

107.Geense WW, van den Boogaard M, van der Hoeven JG, Vermeulen H, Hannink G, Zegers M. Nonpharmacologic Interventions to Prevent or Mitigate Adverse Long-Term Outcomes Among ICU Survivors: A Systematic Review and Meta-Analysis. Critical care medicine. 2019;47(11):1607–18.

108.Cuzco C, Torres-Castro R, Torralba Y, Manzanares I, Munoz-Rey P, Romero-Garcia M, et al. Nursing Interventions for Patient Empowerment during Intensive Care Unit Discharge: A Systematic Review. International journal of environmental research and public health [Internet]. 2021;18(21). Available from: <http://ovidsp.ovid.com/ovidweb.cgi?T=JS&PAGE=reference&D=med20&NEWS=N&AN=34769569>

109.Ridley EJ, Lambell K. Nutrition before, during and after critical illness. Current opinion in critical care. 2022;28(4):395–400.

110.van Zanten ARH, De Waele E, Wischmeyer PE. Nutrition therapy and critical illness: practical guidance for the ICU, post-ICU, and long-term convalescence phases. Critical care (London, England). 2019;23(1):368.

111.Gressies C, Schuetz P. Nutritional issues concerning general medical ward patients: feeding patients recovering from critical illness. Current opinion in clinical nutrition and metabolic care. 2023;26(2):138–45.

112.Pasechnik IN. Nutritional support for critically ill patients (Review). Obshchaya Reanimatologiya. 2020;16(4):40–59.

113.Casaer MP, Ziegler TR. Nutritional support in critical illness and recovery. The lancet Diabetes & endocrinology. 2015;3(9):734–45.

114.Nct. Optimal Timing of Computerized Cognitive Training for Older Intensive Care Unit Survivors. https://clinicaltrials.gov/show/NCT05467410 [Internet]. 2022; Available from: <https://www.cochranelibrary.com/central/doi/10.1002/central/CN-02431092/full>

115.Fadeur M, Preiser JC, Verbrugge AM, Misset B, Rousseau AF. Oral Nutrition during and after Critical Illness: SPICES for Quality of Care! Nutrients [Internet]. 2020;12(11). Available from: <http://ovidsp.ovid.com/ovidweb.cgi?T=JS&PAGE=reference&D=med18&NEWS=N&AN=33202634>

116.Ramsay P, Huby G, Merriweather J, Salisbury L, Rattray J, Griffith D, et al. Patient and carer experience of hospital-based rehabilitation from intensive care to hospital discharge: mixed methods process evaluation of the RECOVER randomised clinical trial. BMJ open. 2016;6(8):e012041.

117.Sjostedt V, Bladh A, Chaboyer W, Johansson L. Patient experiences of an intensive care Liaison Nurse support service. Intensive & critical care nursing. 2022;71:103250.

118.Glimelius Petersson C, Bergbom I, Brodersen K, Ringdal M. Patients’ participation in and evaluation of a follow-up program following intensive care. Acta anaesthesiologica Scandinavica. 2011;55(7):827–34.

119.Plowright C, Fraser J, Smith S, Buras-Rees S, Dennington L, King D, et al. Perceptions of critical care outreach within a network. Nursing times. 2006;102(29):36–40.

120.Nct. Personalized Nutrition Delivery to Improve Resilience in Older Adult Trauma Patients. https://clinicaltrials.gov/ct2/show/NCT05544162 [Internet]. 2022; Available from: <https://www.cochranelibrary.com/central/doi/10.1002/central/CN-02460844/full>

121.Lasiter S, Chrisman M, Snodgrass B, Thompson M, Harmon K. Physical and Cognitive Training to Enhance Intensive Care Unit Survivors’ Cognition: A Mapping Review. Rehabilitation nursing : the official journal of the Association of Rehabilitation Nurses. 2021;46(6):323–32.

122.Griffith DM, Walsh TS. Physical rehabilitation and critical illness. Anaesthesia and Intensive Care Medicine. 2019;20(1):25–8.

123.Connolly B, O’Neill B, Salisbury L, Blackwood B, Enhanced Recovery After Critical Illness Programme G. Physical rehabilitation interventions for adult patients during critical illness: an overview of systematic reviews. Thorax. 2016;71(10):881–90.

124.Connolly B, O’Neill B, Salisbury L, McDowell K, Blackwood B, Enhanced Recovery After Critical Illness Programme G. Physical rehabilitation interventions for adult patients with critical illness across the continuum of recovery: an overview of systematic reviews protocol. Systematic reviews. 2015;4:130.

125.Polastri M. Physiotherapeutic regimen in patients with chronic obstructive pulmonary disease: from the intensive care unit to home-based rehabilitation. International Journal of Therapy & Rehabilitation. 2020;27(1):1–5.

126.Nct. Physiotherapy and Optimised Nutrition in Survivors of Critical Illness. https://clinicaltrials.gov/ct2/show/NCT06159868 [Internet]. 2023; Available from: <https://www.cochranelibrary.com/central/doi/10.1002/central/CN-02632416/full>

127.Svenningsen H, Langhorn L, Agard AS, Dreyer P. Post-ICU symptoms, consequences, and follow-up: an integrative review. Nursing in critical care. 2017;22(4):212–20.

128.Lobo-Valbuena B, Sanchez Roca MD, Regalon Martin MP, Torres Morales J, Varillas Delgado D, Gordo F. Post-Intensive Care syndrome: Ample room for improvement. Data analysis after one year of implementation of a protocol for prevention and management in a second level hospital. Medicina intensiva. 2021;45(8):e43–6.

129.Costa A, Goncalves AF, Rodrigues M, Santos R, Almeida MP, Lima A. Post-intensive Care Unit COVID-19 Survivors: Functional Status and Respiratory Function Three Months After an Inpatient Rehabilitation Program. Cureus. 2022;14(11):e31281.

130.Nienow MK, Susterich CE, Peterson SJ. Prioritizing nutrition during recovery from critical illness. Current opinion in clinical nutrition and metabolic care. 2021;24(2):199–205.

131.Ecklund MM, Bloss JW. Progressive mobility as a team effort in transitional care. Critical care nurse. 2015;35(3):62–8.

132.Wang D, Li J, Zhu F, Hong Q, Zhang M, Gao M, et al. Protocol for a systematic review and meta-analysis of respiratory rehabilitation following intensive care unit discharge for COVID-19 survivors. BMJ open. 2020;10(12):e041184.

133.Ridley EJ, Bailey M, Chapman M, Chapple LAS, Deane AM, Hodgson C, et al. Protocol summary and statistical analysis plan for Intensive Nutrition Therapy comparEd to usual care iN criTically ill adults (INTENT): a phase II randomised controlled trial. BMJ open. 2022;12(3):e050153.

134.Jonasdottir RJ, Jonsdottir H, Gudmundsdottir B, Sigurdsson GH. Psychological recovery after intensive care: Outcomes of a long-term quasi-experimental study of structured nurse-led follow-up. Intensive & critical care nursing. 2018;44:59–66.

135.Blakeney PE, Rosenberg L, Rosenberg M, Faber AW. Psychosocial care of persons with severe burns. Burns : journal of the International Society for Burn Injuries. 2008;34(4):433–40.

136.So Hang M, Yan Wing W, Li Siu C, Leung Yuk W, Shum Hoi P, Lam Sin M, et al. PYICU+ More Is Less: The Post-ICU Discharge Follow-up Programme...20th Congress of Asia Pacific Association of Critical Care Medicine and Annual Scientific Meeting of Hong Kong SCCM 2018, 15-16 December 2018, Hong Kong. Critical Care & Shock. 2019;22(1):64–64.

137.Bloom SL, Stollings JL, Kirkpatrick O, Wang L, Byrne DW, Sevin CM, et al. Randomized Clinical Trial of an ICU Recovery Pilot Program for Survivors of Critical Illness. Critical care medicine. 2019;47(10):1337–45.

138.Gatt M, MacFie J. Randomized clinical trial of gut-specific nutrients in critically ill surgical patients. British Journal of Surgery. 2010;97(11):1629‐1636.

139.Liang Z, Munro C, Yip H, Ji M, Sena Moore K, Ferreira T, et al. Randomized Controlled Study of Self-Managed Music-Guided Exercise Intervention Following Intensive Care. Nursing research. 2023;72(3):193–9.

140.Azoulay E, Vincent JL, Angus DC, Arabi YM, Brochard L, Brett SJ, et al. Recovery after critical illness: putting the puzzle together-a consensus of 29. Critical care (London, England). 2017;21(1):296.

141.Jones C. Recovery post ICU. Intensive & critical care nursing. 2014;30(5):239–45.

142.Nct. REhabilitation After Critical Illness Assisted Discharge Pack (RECAP). https://clinicaltrials.gov/show/NCT02415634 [Internet]. 2015; Available from: <https://www.cochranelibrary.com/central/doi/10.1002/central/CN-01505701/full>

143.Jones C, Skirrow P, Griffiths RD, Humphris GH, Ingleby S, Eddleston J, et al. Rehabilitation after critical illness: a randomized, controlled trial. Critical care medicine. 2003;31(10):2456–61.

144.Salisbury LG, Merriweather JL, Walsh TS. Rehabilitation after critical illness: could a ward-based generic rehabilitation assistant promote recovery? Nursing in critical care. 2010;15(2):57–65.

145.Babu AS. Rehabilitation following return of spontaneous circulation (ROSC-Rehab): A new role for rehabilitation professionals in post-resuscitation care. Resuscitation. 2021;159:38–9.

146.Udina C, Ars J, Morandi A, Vilaro J, Caceres C, Inzitari M. Rehabilitation in adult post-COVID-19 patients in post-acute care with Therapeutic Exercise. The Journal of frailty & aging. 2021;10(3):297–300.

147.Mehlhorn J, Freytag A, Schmidt K, Brunkhorst FM, Graf J, Troitzsch U, et al. Rehabilitation interventions for postintensive care syndrome: a systematic review. Critical care medicine. 2014;42(5):1263–71.

148.Chen DE, Goh SW, Chan HN, Goh HZ, Ong SY, Sim S, et al. Rehabilitation of intubated COVID-19 patients in a Singapore regional hospital with early intensive care unit and sustained post-intensive care unit rehabilitation. Proceedings of Singapore Healthcare [Internet]. 2022;31. Available from: <https://journals.sagepub.com/home/PSH>

149.Goodwin VA, Allan L, Bethel A, Cowley A, Cross JL, Day J, et al. Rehabilitation to enable recovery from COVID-19: a rapid systematic review. Physiotherapy. 2021;111:4–22.

150.Wu TT, Li CX, Zhuang YN, Luo CJ, Chen JM, Li Y, et al. Resistance training combined with β-hydroxy β-methylbutyrate for patients with critical illness: a four-arm, mixed-methods, feasibility randomised controlled trial. Intensive & critical care nursing. 2024;82:103616.

151.Schlitz JM, Fankhauser SL, Tobin AE. Review by the ICU liaison nurse is associated with improved outcomes for patients discharged from ICU with a tracheostomy...The Asia Pacific Critical Care 2008 Congress. Australian Critical Care. 2009;22(1):58–58.

152.Ulutas F, S N Oztekin S, Ardic F. Role of rehabilitation in a COVID-19 survivor with intensive care unit-acquired weakness: A case report. Turkish journal of physical medicine and rehabilitation. 2021;67(1):115–9.

153.Braunschweig CL, Freels S, Sheean PM, Peterson SJ, Perez SG, McKeever L, et al. Role of timing and dose of energy received in patients with acute lung injury on mortality in the Intensive Nutrition in Acute Lung Injury Trial (INTACT): a post hoc analysis. American Journal of Clinical Nutrition. 2017;105(2):411‐416.

154.Tanner J, Cornish J. Routine critical care step-down programmes: Systematic review and meta-analysis. Nursing in critical care. 2021;26(2):118–27.

155.Berney S, Haines K, Skinner EH, Denehy L. Safety and feasibility of an exercise prescription approach to rehabilitation across the continuum of care for survivors of critical illness. Physical therapy. 2012;92(12):1524–35.

156.Jones JRA, Puthucheary Z, McDonald LA, Denehy L, Berney S. Searching for the Responder, Unpacking the Physical Rehabilitation Needs of Critically Ill Adults: A REVIEW. Journal of cardiopulmonary rehabilitation and prevention. 2020;40(6):359–69.

157.Liang Z, Yip H, Sena Moore K, Ferreira T, Ji M, Signorile JF, et al. Self-Managed Music-Guided Exercise Intervention Improved Upper and Lower Extremity Muscle Strength for ICU Survivors-A Pilot Randomized Controlled Study. Biological research for nursing. 2022;24(2):145–51.

158.Vijayaraghavan BKT, Willaert X, Cuthbertson BH. Should ICU clinicians follow patients after ICU discharge? No. Intensive care medicine. 2018;44(9):1542–4.

159.Jones C, Griffiths RD, Skirrow P, Humphris G. Smoking cessation through comprehensive critical care. Intensive care medicine. 2001;27(9):1547‐1549.

160.Vollam S, Efstathiou N. Special issue: Rehabilitation in and after critical care. Nursing in critical care. 2022;27(1):130–2.

161.Morris PE, Berry MJ, Files DC, Thompson JC, Hauser J, Flores L, et al. Standardized Rehabilitation and Hospital Length of Stay Among Patients With Acute Respiratory Failure: A Randomized Clinical Trial. JAMA. 2016;315(24):2694–702.

162.Kwakman RCH, Sommers J, Horn J, Nollet F, Engelbert RHH, van der Schaaf M. Steps to recovery: body weight-supported treadmill training for critically ill patients: a randomized controlled trial. Trials. 2020;21(1):409.

163.Kwakman RCH, Voorn EL, Horn J, Nollet F, Engelbert RHH, Sommers J, et al. Steps to recovery: body weight-supported treadmill training for critically ill patients: a randomized controlled trial. Journal of critical care. 2022;69:154000.

164.Denehy L, Elliott D. Strategies for post ICU rehabilitation. Current opinion in critical care. 2012;18(5):503–8.

165.Jonasdottir RJ, Jones C, Sigurdsson GH, Jonsdottir H. Structured nurse-led follow-up for patients after discharge from the intensive care unit: Prospective quasi-experimental study. Journal of advanced nursing. 2018;74(3):709–23.

166.O’Gara G, Pattison N. Supporting patients recovering after discharge from critical care. Cancer Nursing Practice. 2013;12(1):19–24.

167.Hobson S. Supporting survivorship after critical illness: A service improvement project at a large teaching hospital...Physiotherapy UK Virtual Conference, November 5-6, 2021. Physiotherapy. 2022;114:e198–9.

168.Nct. The CONFUCIUS Oral Protein Supplementation Trial. https://clinicaltrials.gov/show/NCT05405764 [Internet]. 2022; Available from: <https://www.cochranelibrary.com/central/doi/10.1002/central/CN-02405723/full>

169.Salisbury LG, Merriweather JL, Walsh TS. The development and feasibility of a ward-based physiotherapy and nutritional rehabilitation package for people experiencing critical illness. Clinical rehabilitation. 2010;24(6):489–500.

170.Hainsworth T. The development of critical care outreach nursing services. Nursing times. 2006;102(32):25–6.

171.Ali MS, Talwar D, Jain SK. The effect of a short-term pulmonary rehabilitation on exercise capacity and quality of life in patients hospitalised with acute exacerbation of chronic obstructive pulmonary disease. Indian journal of chest diseases & allied sciences. 2014;56(1):13‐19.

172.Lai CC, Chou W, Cheng AC, Chao CM, Cheng KC, Ho CH, et al. The effect of early cardiopulmonary rehabilitation on the outcomes of intensive care unit survivors. Medicine. 2019;98(11):e14877.

173.Wappel S, Tran DH, Wells CL, Verceles AC. The Effect of High Protein and Mobility-Based Rehabilitation on Clinical Outcomes in Survivors of Critical Illness. Respiratory care. 2021;66(1):73–8.

174.Heyland DK, Patel J, Compher C, Rice TW, Bear DE, Lee ZY, et al. The effect of higher protein dosing in critically ill patients with high nutritional risk (EFFORT Protein): an international, multicentre, pragmatic, registry-based randomised trial. Lancet (London, England). 2023;401(10376):568‐576.

175.Tabanejad Z, Pazokian M, Ebadi A. The Effect of Liaison Nurse Service on Patient Outcomes after Discharging From ICU: a Randomized Controlled Trial. Journal of caring sciences. 2016;5(3):215–22.

176.Ataeeara S, Jahani S, Rashidi M, Asadizaker M, Maraghi E, Najafi S. The Effect of Transition Nursing Program from Intensive Care Units to General Units on Anxiety and Satisfaction of Patients and Their Families: A Clinical Trial Study. Journal of Evidence-based Care. 2023;13(4):7–17.

177.Trzmiel T, Marchewka R, Pieczynska A, Zasadzka E, Zubrycki I, Kozak D, et al. The Effect of Using a Rehabilitation Robot for Patients with Post-Coronavirus Disease (COVID-19) Fatigue Syndrome. Sensors (Basel, Switzerland) [Internet]. 2023;23(19). Available from: <http://ovidsp.ovid.com/ovidweb.cgi?T=JS&PAGE=reference&D=med24&NEWS=N&AN=37836950>

178.Nissila E, Hynninen M, Jalkanen V, Kuitunen A, Backlund M, Inkinen O, et al. The effectiveness of a brief intervention for intensive care unit patients with hazardous alcohol use: a randomized controlled trial. Critical care (London, England). 2024;28(1):145.

179.Harrison DA, Gao H, Welch CA, Rowan KM. The effects of critical care outreach services before and after critical care: a matched-cohort analysis. Journal of critical care. 2010;25(2):196–204.

180.Johanna Josepha Op’t Hoog SA, Eskes AM, Johanna van Mersbergen-de Bruin MP, Pelgrim T, van der Hoeven H, Vermeulen H, et al. The effects of intensive care unit-initiated transitional care interventions on elements of post-intensive care syndrome: A systematic review and meta-analysis. Australian critical care : official journal of the Confederation of Australian Critical Care Nurses. 2022;35(3):309–20.

181.Nct. The Energy Dose Study. https://clinicaltrials.gov/show/NCT01369147 [Internet]. 2011; Available from: <https://www.cochranelibrary.com/central/doi/10.1002/central/CN-01591930/full>

182.Molloy J, Pratt N, Reaper S, Dunn E, Botha J, Tobias T. The first 12 months of the critical care liaison nurse service. Australian Critical Care. 2011;24(1):73–73.

183.Ridley EJ, Bailey M, Chapman MJ, Chapple LaS, Deane AM, Gojanovic M, et al. The impact of a tailored nutrition intervention delivered for the duration ofhospitalisation on daily energy delivery for patients with critical illness(INTENT): a phase II randomised controlled trial. Crit Care. 2025;29.

184.Eliott SJ, Ernest D, Doric AG, Page KN, Worrall-Carter LJ, Thalib L, et al. The impact of an ICU liaison nurse service on patient outcomes. Critical care and resuscitation : journal of the Australasian Academy of Critical Care Medicine. 2008;10(4):296–300.

185.Chaboyer W, Gillespie B, Foster M, Kendall M. The impact of an ICU liaison nurse: a case study of ward nurses’ perceptions. Journal of clinical nursing. 2005;14(6):766–75.

186.Neto R, Carvalho M, Paixao AI, Fernandes P, Casteloes P. The Impact of an Intensivist-Led Critical Care Transition Program. Cureus. 2022;14(1):e21313.

187.Rosa RG, Maccari JG, Cremonese RV, Tonietto TF, Cremonese RV, Teixeira C. The impact of critical care transition programs on outcomes after intensive care unit (ICU) discharge: can we get there from here? Journal of thoracic disease. 2016;8(7):1374–6.

188.Lynch J, Cope V, Murray M. The Intensive Care Unit Liaison Nurse and their value in averting clinical deterioration: A qualitative descriptive study. Intensive & critical care nursing. 2021;63:103001.

189.Chaboyer W, Foster MM, Foster M, Kendall E. The Intensive Care Unit liaison nurse: towards a clear role description. Intensive & critical care nursing. 2004;20(2):77–86.

190.Cuthbertson BH, Rattray J, Campbell MK, Gager M, Roughton S, Smith A, et al. The PRaCTICaL study of nurse led, intensive care follow-up programmes for improving long term outcomes from critical illness: a pragmatic randomised controlled trial. BMJ (Clinical research ed). 2009;339:b3723.

191.Pirret AM. The role and effectiveness of a nurse practitioner led critical care outreach service. Intensive & critical care nursing. 2008;24(6):375–82.

192.Aljuhani O. The Role of Critical Care Pharmacists Beyond Intensive Care Units: A Narrative Review on Medication Management for ICU Survivors. Brazilian Journal of Pharmaceutical Sciences. 2022;58:e21012.

193.Nct. The Role of NEMS for Post ICU Rehabilitation. https://clinicaltrials.gov/show/NCT01717833 [Internet]. 2012; Available from: <https://www.cochranelibrary.com/central/doi/10.1002/central/CN-02028767/full>

194.Moisey LL, Merriweather JL, Drover JW. The role of nutrition rehabilitation in the recovery of survivors of critical illness: underrecognized and underappreciated. Critical care (London, England). 2022;26(1):270.

195.Bear DE, Wandrag L, Merriweather JL, Connolly B, Hart N, Grocott MPW, et al. The role of nutritional support in the physical and functional recovery of critically ill patients: a narrative review. Critical care (London, England). 2017;21(1):226.

196.Antonnacci R, Sanzone L, Gauthier M, Di Giovanni C, Krishnapillai N, Usuike M. The Use of the ABCDEF Bundle to Promote Interprofessional Communication and Continuity of Post-Intensive Care Syndrome (PICS) Prevention in Post-intensive Care Unit (ICU) Settings...Dynamics of Critical Care Conference, September 28–30, 2020, Windsor, Ontario. Canadian Journal of Critical Care Nursing. 2020;31(1):25–25.

197.Carvalho AC, Moreira J, Cubelo P, Cantista P, Branco CA, Guimaraes B. Therapeutic respiratory and functional rehabilitation protocol for intensive care unit patients affected by COVID-19: a structured summary of a study protocol for a randomised controlled trial. Trials. 2021;22(1):268.

198.Parker V, Giles M, Shylan G, Austin N, Smith K, Morison J, et al. Tracheostomy management in acute care facilities--a matter of teamwork. Journal of clinical nursing. 2010;19(9–10):1275–83.

199.Haruna J, Masuda Y, Tatsumi H. Transitional Care Programs for Patients with High Nursing Activity Scores Reduce Unplanned Readmissions to Intensive Care Units. Medicina (Kaunas, Lithuania) [Internet]. 2022;58(11). Available from: <http://ovidsp.ovid.com/ovidweb.cgi?T=JS&PAGE=reference&D=med22&NEWS=N&AN=36363489>

200.Nct. Treatment of Critical Illness Polyneuromyopathy. https://clinicaltrials.gov/show/NCT01058421 [Internet]. 2010; Available from: <https://www.cochranelibrary.com/central/doi/10.1002/central/CN-02035427/full>

201.Australian ICULNF. Uptake and caseload of intensive care unit liaison nurse services in Australia. Critical care and resuscitation : journal of the Australasian Academy of Critical Care Medicine. 2012;14(3):221–6.

202.Haggstrom M, Fjellner C, Ohman M, Rising Holmstrom M. Ward visits- one essential step in intensive care follow-up. An interview study with critical care nurses’ and ward nurses’. Intensive & critical care nursing. 2018;49:21–7.
